# Printed temperature sensor array for high-resolution thermal mapping

**DOI:** 10.1038/s41598-022-18321-6

**Published:** 2022-08-20

**Authors:** Tim Bücher, Robert Huber, Carsten Eschenbaum, Adrian Mertens, Uli Lemmer, Hussam Amrouch

**Affiliations:** 1 University of Stuttgart, Semiconductor Test and Reliability (STAR), Pfaffenwaldring 47, 70569 Stuttgart, Germany; 2 Karlsruhe Institute for Technology (KIT), Light Technology Institute (LTI), Engesserstrasse 13, 76131 Karlsruhe, Germany; 3 Karlsruhe Institute for Technology (KIT), Institute of Photonics and Quantum Electronics (IPQ), Engesserstrasse 5, 76131 Karlsruhe, Germany

**Keywords:** Engineering, Electrical and electronic engineering

## Abstract

Fully-printed temperature sensor arrays—based on a flexible substrate and featuring a high spatial-temperature resolution—are immensely advantageous across a host of disciplines. These range from healthcare, quality and environmental monitoring to emerging technologies, such as artificial skins in soft robotics. Other noteworthy applications extend to the fields of power electronics and microelectronics, particularly thermal management for multi-core processor chips. However, the scope of temperature sensors is currently hindered by costly and complex manufacturing processes. Meanwhile, printed versions are rife with challenges pertaining to array size and sensor density. In this paper, we present a passive matrix sensor design consisting of two separate silver electrodes that sandwich one layer of sensing material, composed of poly(3,4-ethylenedioxythiophene):polystyrene sulfonate (PEDOT:PSS). This results in appreciably high sensor densities of 100 sensor pixels per cm$^2$ for spatial-temperature readings, while a small array size is maintained. Thus, a major impediment to the expansive application of these sensors is efficiently resolved. To realize fast and accurate interpretation of the sensor data, a neural network (NN) is trained and employed for temperature predictions. This successfully accounts for potential crosstalk between adjacent sensors. The spatial-temperature resolution is investigated with a specially-printed silver micro-heater structure. Ultimately, a fairly high spatial temperature prediction accuracy of 1.22  °C is attained.

## Introduction

With the advent of printable sensors, conventional electronics are now equipped with a new generation of sensors that feature high adaptability and mechanical flexibility. These instrumental properties of printed sensors add further value to a variety of applications—in healthcare^1–6^, robotics^7–9^, environmental surveillance^10–13^ and quality assurance in the food industry^14–16^. In particular, for microelectronic applications, obtaining high-resolution thermal maps of Integrated Circuits (ICs) empowers the developers as well as the operating system to make better design-time and run-time decisions, especially with regard to managing the reliability and temperature of many-core processor chips^17–19^. Most methods utilize either the resistance changes of conductors or the Seebeck effect of material combinations. Additionally, a multitude of fabrication techniques have been pioneered over the past few years. Chief among them are printing techniques such as inkjet^20–22^ and screen printing^20,23,24^. Inkjet printing has become particularly prominent over the past decade. This can be mainly attributed to a combination of: (a) rapid and maskless prototyping, (b) conservative ink consumption and (c) steadily increasing number of available inks^22^. Screen printing is a fully-fledged industrial printing process. This approach benefits from consistently reproducible and predictable results, offering high throughput even with large-scale samples^23^. With regard to the sensing material, various approaches have proven suitable for printed temperature sensors. Among them, carbon nanotubes (CNTs)^8,25^, metal nanowires/nanoparticles^5,26,27^, graphene^2,28^ and different polymers^29,30^ have been reported. One prominent polymeric example is PEDOT:PSS. Several properties make this polymer an interesting and promising sensing material because it can be modified and specially treated to achieve high mechanical stability and electrical tunability^4,31,32^. Meanwhile, it remains user-friendly and compatible with various printing processes. In practice, PEDOT:PSS is neither toxic nor water pollutant, and therefore, appealing for many applications. The PEDOT:PSS inks can also be adapted to specific printing technologies through the use of additives^33,34^.

Depending on the field of application and measurement method, various sensor design approaches have been proposed in literature. These range from single sensors on a small-scale^9^ to multiple sensors combined on a larger sensor array^20,29,35^. One simplistic approach is a meander structure^36,37^, usually printed with metal inks. This simultaneously functions as an electrode and a sensing layer. Other designs use several materials and measure the lateral resistance change of the sensing material between metallic finger structures^4^. Printable and flexible devices have proven to be consequential for rough measurement conditions with high deformation tolerances. Printed thermocouples^9,38,39^, comprised of two materials, deliver promising results with high accuracy and stability. Nevertheless, most of the proposed sensors rely on the thermistor principle, capable of achieving a high electrical resistance change due to the undergoing change in temperature. This is paramount to ensure reliable reading from the peripheral readout circuits. In this case, research is being conducted into materials that have a high temperature coefficient of resistance (TCR) and high resistance, but at the same time cover a wide temperature range and allow easy processing during manufacturing. When dealing with non-ideal material properties this can be compensated for by a improved sensor structure or by a adapted readout electronic. However, it results in larger sensor areas and/or complex sensor layouts incorporating active components.

**Figure 1:**
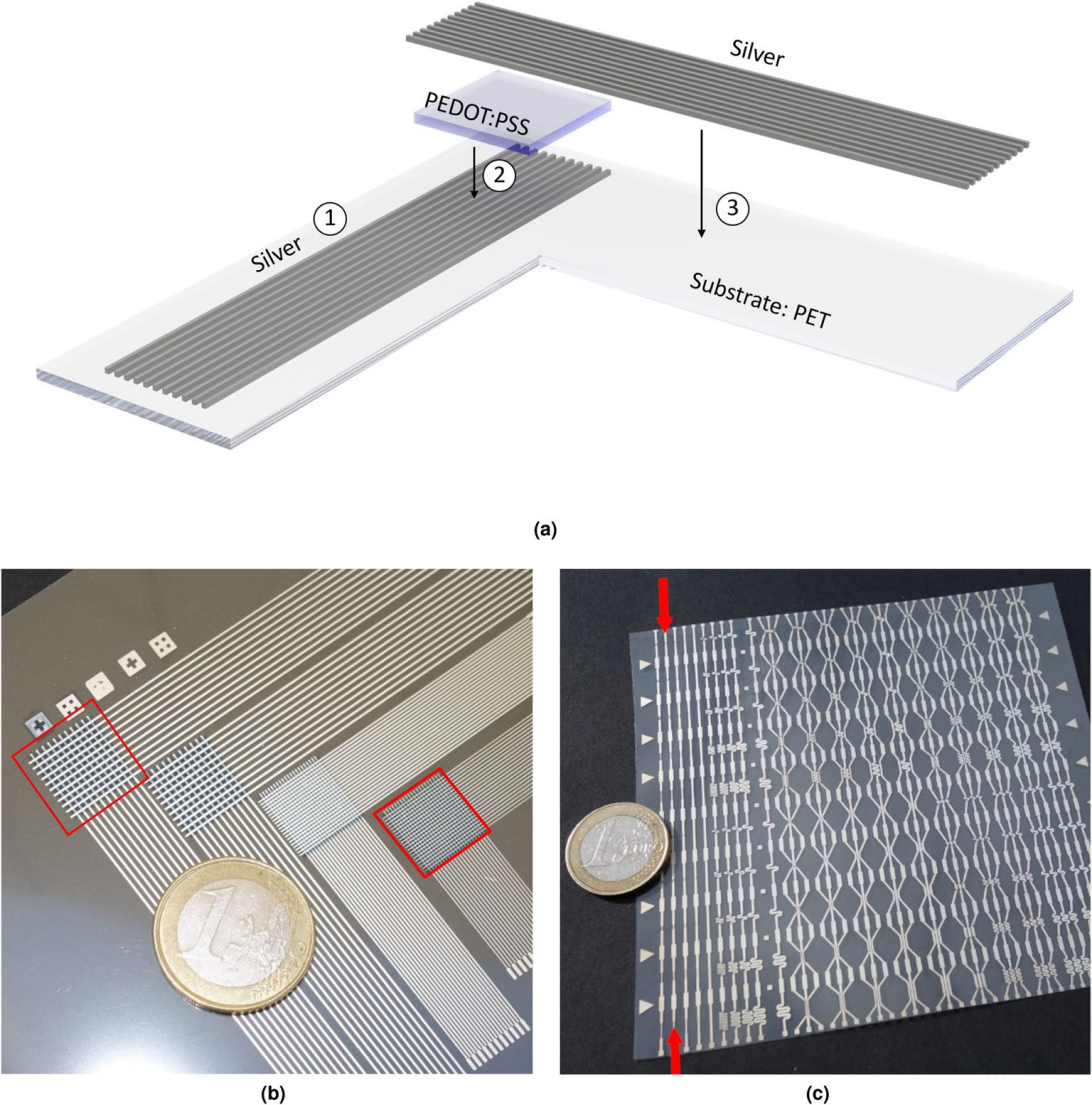
(**a**) Layer structure of the temperature sensor. The measuring principle is based on a passive matrix with a 10 × 10 electrode structure. Three different layers are consecutively deposited on a PET substrate using a screen printing process: (1) Silver bottom electrode, (2) multiple PEDOT:PSS-layers to increase thickness, (3) Silver top electrode. (**b**) Fully printed sensors with different silver line width. Two printed temperature array examples are highlighted demonstrating the capability of our approach to achieve a high number of printed sensors within a small area footprint (i.e., a high sensors density of $11 \times 11 = 121$ within 1 cm$^2$ and $21 \times 21 = 441$ within 1 cm$^2$). It is noteworthy that the sensor with the lowest sensor density was chosen to demonstrate the results, due to limitation of the readout electronics and for a favorable higher readout speed. (**c**) Different heater designs incorporated in one print layout.

In this work, we present a novel fully-screen printed passive matrix temperature sensor based on the sensor material PEDOT:PSS. The latter is embedded between two silver electrodes. Our sandwich design allows for a high sensor density of 100 sensor pixels covering an area of 1 cm^2^. The sensors were successfully and reliably operated between $20$ °C and $90$ °C. The combination of these two properties makes our temperature sensor array enticing, not only for healthcare applications, robotics and electronic skins but also for temperature monitoring of electronic devices and ICs, especially processor chips. The sensor array (see Fig. 1) is printed on a flexible 100 μm-thick polyethylene terephthalate (PET) substrate, making it easy applicable for different use cases and surfaces shapes, i.e. following the curvature of a fingertip and providing the flexibility to bend the finger. The used silver convinces with good printabilty, high conductivity and scratch resistance. PEDOT:PSS is a well known material offering the above-mentioned advantages. Finally, the sensor effectively demonstrates the spatio-temporal capture of the temperature evolution. It is noteworthy that the term “high resolution” in the context of this work refers to the *spatial temperature resolution*. In other words, the high achieved sensor density (i.e., high number of printed thermal sensors within a small area footprint). A Machine Learning (ML) approach using a small NN is adopted to “learn” the sensors’ behavior, wherein resistance changes of the sensors are carefully correlated with the corresponding temperature. Because this is done for each sensor pixel individually, variations from fabrication can be compensated.

## Results

**Figure 2:**
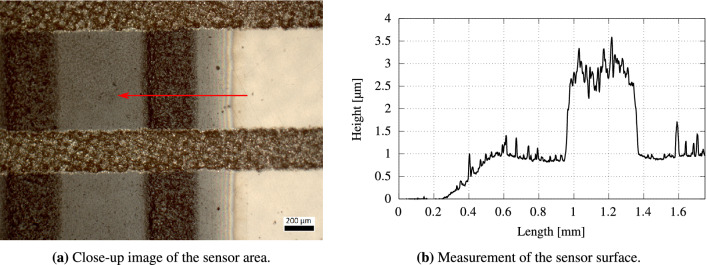
(**a**) Close-up image of the temperature sensor array with the bottom electrode (vertical lines), top electrode (horizontal lines) and the sandwiched PEDOT:PSS layer (transparent blue). The red arrow depicts the measurement direction conducted with a profilometer: (**b**) Surface structure of the sensor area. The PEDOT:PSS layer has a thickness of about 1 $\upmu$m. The underlying silver electrode has a thickness of 2–3 $\upmu$m. This thickness is found equal for top and bottom electrodes.

The design and fabrication steps of the temperature sensor are shown in Fig. 1a and the fully fabricated sensor is shown in Fig. 1b, including a close-up image of one sensor pixel in Fig. 2a. Further, Fig. 1c demonstrates the used layout that incorporates several heater designs with different spatial patterns, which is later used for evaluating the behaviour of the printed thermal sensors.

**Figure 3:**
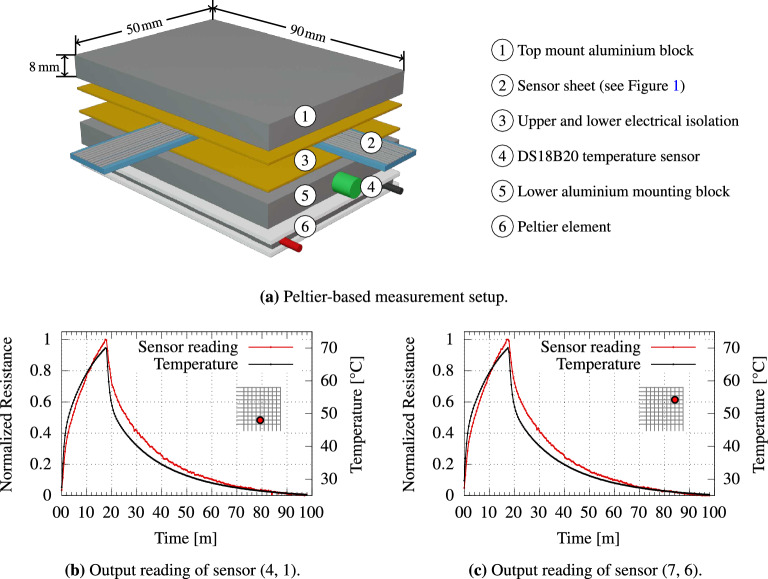
(**a**) The peltier-based measurement setup. (**b**,**c**) In a first step the peltier element is set to reach a temperature of  $70$ °C. After the heating phase, the peltier element is turned off, which allows the sensor array to cool down. During the heating and cooling periods, the sensor array sheet is continuously read. Two examples for the obtained sensor readings along with the corresponding temperature for two different temperature sensors within the array are presented in (**b**,**c**). As shown, the changes in the resistance of the PEDOT-based temperature sensor follows the changes of the temperature.

Figure 3a depicts a custom-built Peltier-based measurement setup; Fig. 3b,c demonstrate the typical response observed for heating the Peltier element to $70$ °C, and the subsequent cooling phase by simply turning it off. During the heating and cooling periods, the sensor array sheet is continuously read. Two examples for the obtained sensor readings along with the corresponding temperature for two different temperature sensors within the array are presented in Fig. 3b,c. As shown, the changes in the resistance of the PEDOT-based temperature sensor closely follow the reading of the reference sensor.

**Figure 4:**
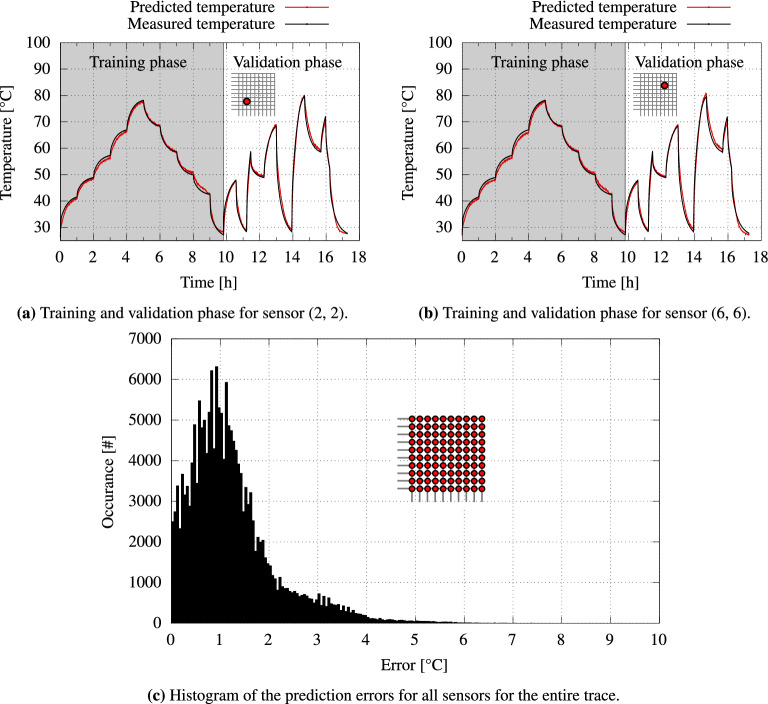
(**a**,**b**) Example of the training and validation phases for two different exemplary temperature sensors across the array. (**c**) The histogram presents the distribution of temperature prediction error for all sensors during the entire validation phase.

Each sensor array pixel might have a slightly different behavior i.e., different temperature dependency. This is an unavoidable consequence of the printing process and potential crosstalk between adjacent sensor pixels. To account for such inherent variations and guarantee accurate temperature predictions, we employ a machine learning method that trains a small NN. The NN captures i.e., “learns” the behavior of the printed sensors to perform the required temperature predictions. During the NN training phase (see Fig. 4a,b), the voltage supplied to the Peltier element is increased step-wise to a maximum temperature of $90$ °C. The supply voltage is then gradually decreased until the starting temperature of $25$ °C is reached again. During the training phase, the temperature sensor array is read continuously, and the collective sensor data are recorded along with the corresponding measured reference temperatures. Subsequently, this data is used to train a small NN that eventually performs the required temperature predictions. The trained NN model can predict the temperature separately for each sensor (see right-hand side of Fig. 4a,b). To this end, a small fully-connected NN is trained and utilized for inference, to translate the sensor reading to the respective temperature. As inputs, the network receives: (a) a three-dimensional vector consisting of the raw sensor reading *r* and (b) the column and row number of the corresponding sensor. The network proceeds to output the temperature *T* of the location indicated by *row* and *col*. Sensors within the same array and/or different printed arrays might have slightly different resistance characteristics, due to inherent variations during the printing process. Updating the ML model with new data from the respective sensor successfully captures these characteristics, thereby accounting for inherent variations. A histogram reporting the prediction error is presented in Fig. 4c. The histogram comprises the prediction errors of all the sensors throughout the validation phase. The average error in temperature prediction is around $1.22$ °C, with a variance ($\sigma ^2$) of 0.77 K$^2$. Experimental results further demonstrate that the entire sensor array can be fully read, and the corresponding temperature predictions are inferred within 27 s. This includes the time overheads of the periphery readout circuits for sampling and multiplexing, as well as the NN inference time taken to predict the corresponding temperature values of all sensor readings. A standalone sensor reading can be obtained within 90 ms. Further, the mentioned value of $1.22$ °C in our work refers to the accuracy of the trained NN when predicting/inferring the temperature of a single pixel from the obtained readout. The readout circuit is capable of resolving temperature with a resolution of $0.02$ °C. Hence, taking the presented results, utilizing a well trained NN provides fast yet accurate temperature predictions during the inference.

**Figure 5:**
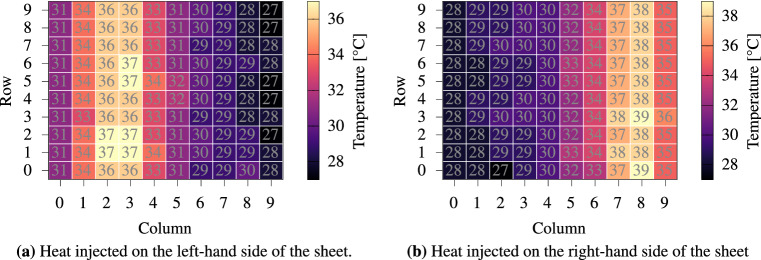
By using the micro heater sheet (Fig. 1c), we are able to inject heat in arbitrary regions of the sensor sheet.

To investigate whether the sensor array can correctly detect spatial-temperature distributions, heat is induced in different regions throughout the sensor sheet using the printed micro-heaters, shown in Fig. 1c. The measurement setup is modified such that the printed micro-heater sheet is incorporated between the temperature sensor sheet and lower electrical isolation (Fig. 3a (2) and (3), respectively). Figure 5a,b shows the thermal maps captured after inferring the temperature for each sensor on the sensor array.

**Figure 6:**
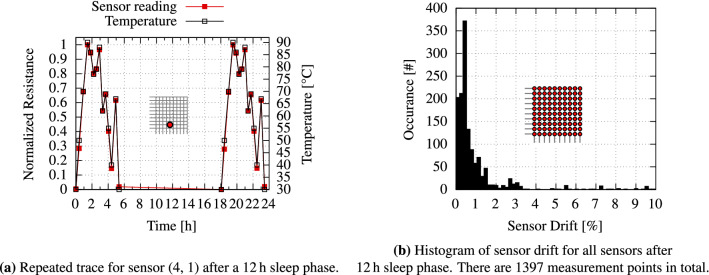
The sensor sheet is mounted between the two aluminum blocks and placed inside a thermal chamber. Subsequent temperatures are reached and the sensor sheet is read at each point.

To further validate the functionality of the temperature sensor, the setup is transferred to a thermal chamber with a controlled environment, instead of introducing heat to the sensor array with a Peltier element. Such an environment offers the dual benefits of maintaining a stable temperature for an extended period of time. The sensor sheet is mounted between two aluminum blocks separated by an isolation layer that prevents short circuits. It is then placed within the thermal chamber (“Vötsch VT4002”) together with the measurement circuit. The Raspberry Pi that modulates the measurement circuit is placed outside the chamber. It controls the thermal chamber via an Ethernet connection to request and collect the desired temperature traces. Figure 6a shows the long-term trace of a sensor on a sensor sheet, and the corresponding thermal chamber temperature. To ensure temporal stability of the temperature sensors and analyze any potential drift in the PEDOT:PSS-material (i.e., resistance-temperature correlations) with time, measurements were repeated after a prolonged period of “sleep,” spanning 12 h. During this period, environmental conditions such as temperature were kept constant within the thermal chamber, even so the thermal chamber is not capable of controlling the humidity. Analyzing the impact of humidity condition is interesting but it goes beyond the scope of this work. Figure 6b, which quantifies the error caused by potential sensor drift, depicts a good correspondence between sensor reading and chamber temperature. Notably, this trend persists even after the “sleeping” interval. The thermal sensing capability of the temperature sensors is governed by the temperature coefficient of resistance (TCR) of the material, which defines the sensitivity of the temperature sensor. It can be calculated by Eq. (1):

1 $$\begin{aligned} TCR = \frac{R-R_0}{R_0} \times \frac{1}{\Delta T} \times 100 \end{aligned}$$

**Figure 7:**
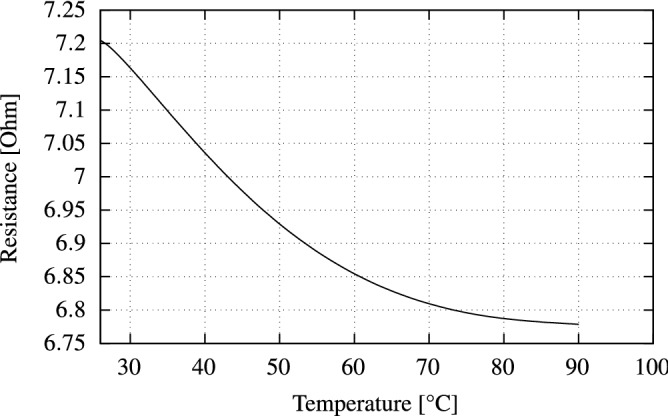
Resistance of the PEDOT:PSS layer at a temperature range from $25$ to $90$ °C.

Here, *R* denotes the resistance at the highest temperature while $R_0$ denotes the starting resistance, e.g. at room temperature ($25$ °C). $\Delta T$ describes the change in temperature. Using this formula, we calculate the TCR of the proposed temperature sensor to be ${0.09}~\%\text {K}^{-1}$ for temperature sensing between $25$ and $90$ °C (see Fig. 7).

## Discussion

**Figure 8:**
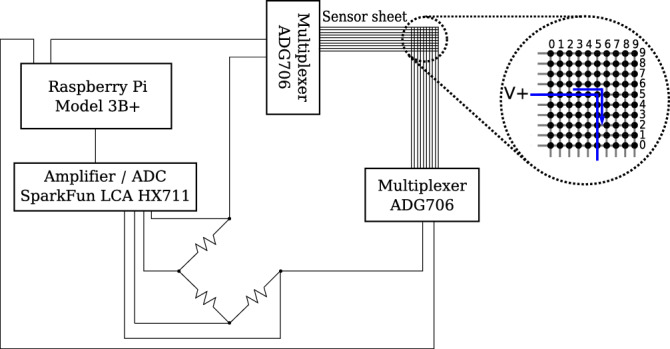
The silver electrode forms a regular grid layout. When measuring a specific point on the sensor sheet, a voltage is applied to a given row and ground is connected to the corresponding column, allowing a current to flow. The PEDOT:PSS layer causes a temperature dependant voltage drop which can be used to reconstruct the temperature at this point.

The electrical properties of the PEDOT:PSS layer allow for its application as a thermistor, as shown by previous work^34^. The sensor composition of PEDOT:PSS and silver show antagonist TCR behavior, which is dominated by the temperature-dependent NTC variations in electrical resistance of the PEDOT:PSS-layer. We exploit this property by sandwiching a PEDOT:PSS layer between two layers of straight-lined silver electrodes at a $90^\circ$ angle, forming a regular grid as shown in Fig. 8. Each individual junction in this grid can be viewed as a single thermistor at that specific point. An electrical current flows when voltage is applied to a given row, and the ground is connected to the corresponding column (Fig. 8). The electrical resistance of the PEDOT:PSS layer at each pixel is dependent on the temperature at that specific point. Using this dependency, we can measure the voltage drop at each pixel. In the NN training phase, we repeat the aforementioned step for each sensor across a series of fixed temperatures. This helps to correlate the resistance at each point to a known temperature.

**Figure 9:**
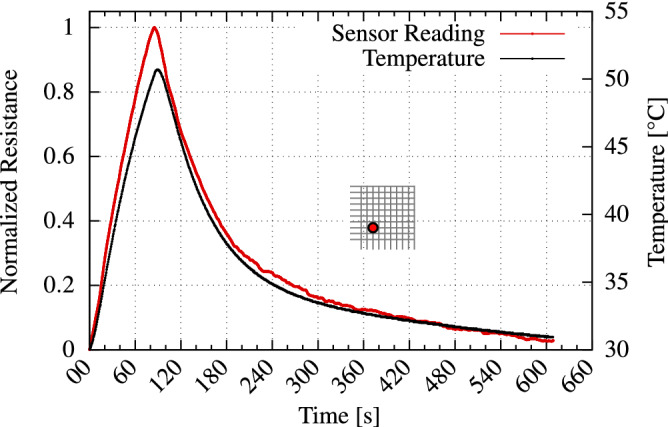
Output reading of sensor (2, 2) sampled at highest possible frequency. When sampling only a single sensor, thus avoiding all multiplexing and stabilization overhead, a high temporal resolution of 90 ms can be achieved.

In the following, we discuss the different types of latency that can be incurred during the measurement sequence. (1) Expected measurement latency: The required time for reading a specific sensor is 270 ms, on average. This includes the time that the readout circuitry (see Fig.  8) may require to stabilize, thus providing reliable readings with appreciable certainty. Taking into account the additional multiplexing delay (see Fig. 8), 27 s to 28 s are required to read the entire sensor array. When sampling a single sensor continuously (see Fig. 9), the multiplexing and stabilization overhead are absent, thereby reducing the sensor readout time to 90 ms. (2) Inference latency for temperature prediction: Using the trained NN model, inferring the temperature of a single sensor, specified by its row and column coordinates on the sensor sheet, takes on average 24 ms. Due to vector processing, obtaining the temperature for the entire sensor array contributes only marginally to the total delay and requires 38 ms on average. Therefore, data acquisition from the sensor array can be continued seamlessly. Moreover, the inference for the entire array from the previous measurement iteration can be completed in the background.

## Methods

### Sensor array fabrication

The temperature sensor’s design and fabrication steps are shown in Fig. 1a and the fully fabricated sensor is presented in Fig. 1b. All the samples are prepared on flexible polyethylene terephthalate (PET) substrates of 100 $\upmu$m-thickness which are rinsed with 2-propanol and dried with an air gun. The fabrication procedure is comprised of three printing steps, each of which prints a layer with an individual screen. A silver paste is used for both the top and bottom electrode layers. The active layer of the thermistor is deposited by the multipass printing of PEDOT:PSS-layers. All five PEDOT:PSS-layers total about 1 $\upmu$m in thickness. Given the necessity for multiple layers, perfect layer matching is ascertained through alignment marks on each printed layer. The subsequent screen printing process is performed on a semi-automatic screen printing machine (Kammann K15 Q-SL) within three printing steps, with an individual screen for each layer. As shown in Fig. 1a-1, the base electrode is printed with a commercial silver paste (Loctite ECI 1010 E &C, Henkel) using a screen fineness of 420 threads/inch (165 threads/cm) and a thread diameter of 27 $\upmu$m. Subsequently, the silver layer is cured on a hotplate at 120 °C for 15 min, finally producing a layer thickness of approximately 3 $\upmu$m and resulting in a resistance of about 19.6 $\Omega$. The overall length is 10 mm with a line width of 400 $\upmu$m. In the succeeding step, five individual PEDOT:PSS-layers (Clevios S V4, Heraeus) (Fig. 1a-2) are printed atop the first silver electrode. Each layer is dried on a hotplate at 120 °C for 10 min before the next layer is deposited. The screen for PEDOT:PSS features a fineness of 350 threads/inch (140 threads/cm) and a thread diameter of 31 $\upmu$m. All five PEDOT:PSS-layers sum up to about 1 $\upmu$m thickness. Lastly, the top silver electrode (Fig. 1a-3) is printed with the same parameter sets as the base electrode. All film thicknesses are measured with a tactile stylus profiler (Dektak XT, Bruker). A close-up image and the surface measurement of the sensor structure are shown in Fig. 2 Due to the necessity for multiple layers, special alignment marks are included on each printed layer to ensure perfect layer matching. The fully fabricated sensor is shown in Fig. 1b.

In addition to the intended low complexity, the presented sensor layout offers a high error tolerance for the layer alignment during the printing process. The sensor pixels are defined by the intersecting silver electrodes with the intermediate PEDOT:PSS layer. Accordingly, it must be ensured that all electrodes overlap. Given the overall size, this is easy to carry-out. The intermediate thermistor pad size is chosen larger than the dimensions of the electrode grid, providing more space for alignment. Additionally, we added several markers at the layout edges, which the layers can be easily aligned to. In practice, we first printed each layer onto a attached paper sheet, which afterwards perfectly represented the alignment of the printing screen. This was only necessary for the second and third layer, where the previously deposited layer had to be aligned to next layer. With the taken measures, we did not observe any problems attributed by misalignment.

### Micro heater fabrication and characterization

The micro heater used for testing the spatial resolution of the sensor array consists of a flexible 100 $\upmu$m-thick polyethylene terephthalate (PET) substrate, with one printed silver layer. The printing process is similar to that of the temperature sensor’s base silver electrode. A layer thickness of about 4 $\upmu$m is used. Our layout incorporates several heater designs with different spatial patterns, depicted in (Fig. 1c).

**Figure 10:**
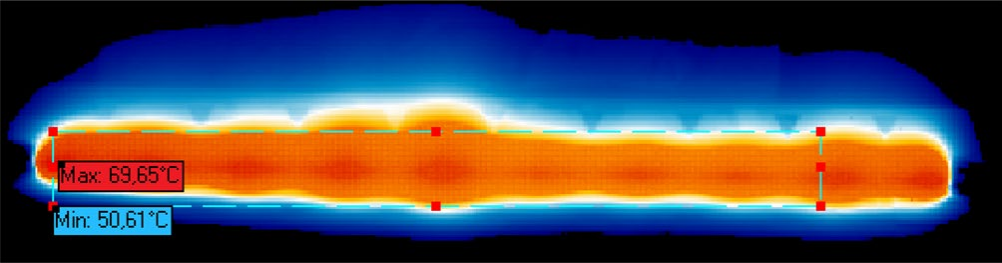
Infrared image of a printed micro heater during operation.

**Figure 11:**
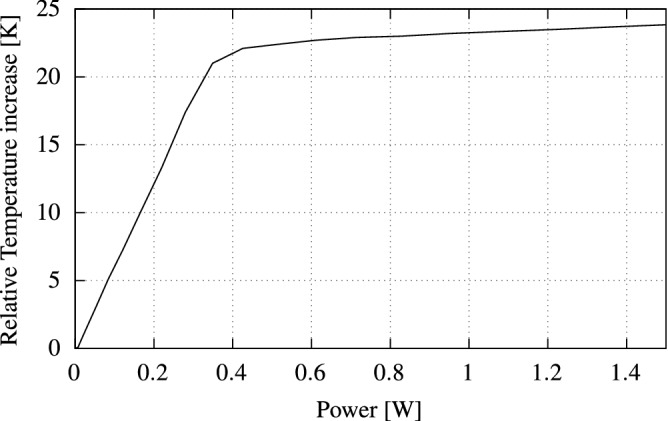
Temperature response of the printed micro heaters for a given power.

To accurately characterize the printed micro heaters, we employ an ImageIR 8300 infrared (IR) camera^40^ to capture the resulting temperature response of the printed micro heater at a given input power. This indium antimonide (InSb)-based IR camera provides a measurement accuracy of $\pm \,{1}\,^\circ \text {C}$ with a temperature resolution of ${0.025}\,\text {K}$. Figure 10 presents an example of the captured thermal image. The micro heaters surface has been covered with a black tape in order to achieve a known emissivity. This allows the IR camera software to perform an internal calibration and compensate any potential loss in the readings accuracy. The current supplied to the micro heater was steadily increased from ${0}\,\text {mA}$ all the way up to ${400}\,\text {mA}$ in steps of ${20}\,\text {mA}$. The resulting voltage has been recorded and the total power consumption was then calculated. Figure 11 demonstrates the relative temperature increase of the printed micro heaters as function of consumed power.

### Built setup for measurement and evaluation

The measurement setup of Fig. 3a, was assembled to validate the general functionality of the printed sensors. It consists of the temperature sensor array and the periphery circuit, combined with a thermoelectric (Peltier) element. By modulating its input voltage, the Peltier element allows us to generate controlled temperature traces by heating and cooling the sensor array. This consequently makes it possible to monitor and record the response of the temperature sensors. Proper thermal conductivity and electrical isolation are assured by sandwiching the temperature sensor array between two isolating layers of electrical tape. This further prevents short circuits via the top and/or bottom aluminum plates. To obtain a reference temperature, an external temperature sensor (DS18B20, Maxim Integrated) is placed deep within the lower aluminum mount plate. Thermal conductivity is enhanced by filling the narrow, empty space between external temperature sensor and the surrounding aluminum with a thin film of thermal paste (i.e., thermal interface material). The entire setup is controlled by a Raspberry Pi Model 3B+ that regulates the power supply of the Peltier element, monitors and records the reference temperature and extracts all measurement data of the temperature sensor array. The details about the dimensions of components used in the evaluation setup are presented in Table 1. Note that the silver electrode forms a regular grid layout. When measuring a specific point on the sensor sheet, a voltage is applied to a given row and ground is connected to the corresponding column, allowing a current to flow. The PEDOT:PSS layer causes a temperature dependant voltage drop which can be used to reconstruct the temperature at this point.

**Table 1:** Dimensions of the components of the evaluation setup shown in Fig. 3.

Component	Width $[\text {mm}]$	Length $[\text {mm}]$	Height $[\text {mm}]$
➀	50	90	8
➂	50	90	2
➄	50	90	8
➅	50	90	4

### Data acquisition

The data acquisition circuit is illustrated in Fig. 8. Each row and column of the temperature sensor array sheet connects to a multiplexer via a Flexible Printed Circuit (FPC) connector. The overall setup is constituted by two FPC connectors (one for the rows and one for the columns). Each FPC is connected to an analog, low resistance (2.5 $\Omega$) multiplexer. Such an arrangement makes it possible to select each individual sensor to be measured across the array. The analog output of the multiplexers is then passed to a *Wheatstone bridge* style circuit. Here, it is amplified and digitized by an integrated HX711 load cell amplifier using a 24-bit Analog-Digital Converter (ADC). The entire measurement circuit is managed by a Raspberry Pi Model 3B+ that controls the multiplexing, timing, amplifier read-out and data collection.

### Neural network for temperature prediction

The NN was implemented in Python 3 using *scikit-learn* library^41^ and consists of four layers: an input layer, two hidden layers and an output layer. Each hidden layer consists of 15 neurons, resulting in a total of 285 weights. The individual neurons are activated by a *sigmoid* function, $\frac{1}{1+e^{-x}}$ and the gradient descent algorithm used is *adam*^42^.

As shown in Fig. 4a, we continuously record all readings from the temperature sensor array while simultaneously keeping track of the current reference temperature. The collected data during this learning phase of the NN is then split as follows: 75% for training, 15% for validation and 10% for testing data. On average, training the NN takes 32 minutes on an Intel Core i3-2100 CPU at 3.10 GHz with 8 GB of memory to reach an $R^2$ score of 0.96. Noteworthily, training is a one-time effort during the design time.

## Summary and conclusions

In this work, we demonstrated the first temperature sensor array comprising $100+$ sensors within a small footprint (merely 1 cm$^2$), which is fully printed in a non-complex manner. The achieved sensor density and hence the provided spatial thermal resolution is unmatched for fully printed temperature sensor arrays compared to state of the art (see Table 2). To keep the complexity low, we relied on a well-established and simple deposition methodology, such as screen printing, combined with commercially available ready-to-use inks. The passive matrix structure fulfills the simplicity requirements and consists of only three layers. We believe that the simplicity is a key for such an approach to be adopted. This makes the fabrication process as well as the alignment process and the sensor’s behaviour itself less error prone, highly cost efficient and easily scalable. To measure and characterize the sensors array, a special self-sufficient readout unit was developed. Then, a ML-based approach was implemented on the readout unit capable of compensating fluctuation of the sensor sheet and making the setup robust in which a fairly high temperature prediction accuracy of $1.22$ °C is attained. The ML-based approach allows us to additionally account for potential crosstalk between adjacent sensors.

**Table 2:** Overview and comparison against the existing state-of the-art printed temperature sensor arrays.

Reference	Fabrication $\left[ \text {fully printed}\right]$	Material	Size $\left[ \mathrm {cm}^2\right]$	Sensors $\left[ \#\right]$	Density $\left[ \#/\mathrm {cm}^2\right]$	Pixel size $\left[ \mathrm {mm}^2\right]$	Temperature range $\left[ ^{\circ }\text {C}\right]$	TCR $\left[ \%/\text {K}\right]$
^29^	Screen printing $\left[ \mathrm {yes}\right]$	NTC ceramic composite	7 × 13	414	4.5	6 × 6	40–140	4.00
^33^	Inkjet/Screen printing $\left[ \mathrm {yes}\right]$	Graphene PEDOT:PSS	2 × 3	4	0.7	1 × 1	35–45	0.06
^4^	Inkjet $\left[ \mathrm {yes}\right]$	PEDOT:PSS CYTOP	1 × 1	1	1.0	5 × 5	25–50	0.77
^8^	Screen printing $\left[ \mathrm {yes}\right]$	CNT PEDOT:PSS	8 × 8	9	0.2	2 × 4	20–80	0.25
^17^	Thermal evaporation $\left[ \mathrm {no}\right]$	Pentacene Ag NPs	4 × 4	256	16.0	2.5 × 2.5	20–100	< 0.04
^43^	Dispenser $\left[ \mathrm {no}\right]$	Graphite PDMS	4 × 4	64	5.3	1.5 × 1.5	30–110	< 0.29
^44^	VLSI $\left[ \mathrm {no}\right]$	Silicon	0.8 × 0.8	1024	1600	0.05 × 0.05	30–110	< 0.29
^45^	Masking $\left[ \mathrm {no}\right]$	CNT PEDOT:PSS	8 × 8	12	5.3	2 × 4	20–60	0.40
^46^	Masking $\left[ \mathrm {no}\right]$	Graphite copolymer	3.8 × 3.8	144	10.2	2 × 2	25–50	NA
This work	Screen printing $\left[ \mathrm {yes}\right]$	PEDOT:PSS	1 × 1	121 (up to 441)	121 (up to 441)	0.4 × 0.4 (down to 0.1 × 0.1)	20–90	0.09

## Data Availability

The datasets used and/or analyzed during the current study available from the corresponding author on reasonable request.
